# The impact of spatial skills on problem‐solving parsimony and solution quality in middle childhood

**DOI:** 10.1111/bjdp.12568

**Published:** 2025-05-27

**Authors:** Jonas Schäfer, Timo Reuter, Miriam Leuchter

**Affiliations:** ^1^ Department of Pedagogical Psychology and Health Psychology University of Education Schwäbisch Gmünd Schwäbisch Gmünd Germany; ^2^ Institute for Child and Youth Education University of Kaiserslautern‐Landau (RPTU) Landau Germany

**Keywords:** cognitive development, mental rotation, spatial skills, STEM performance, visuo‐spatial memory

## Abstract

Spatial skills are essential cognitive abilities that develop during middle childhood and play a crucial role in solving STEM problems. In this relation, however, important aspects of problem‐solving performance remain underexplored. Consequently, this study investigated whether spatial skills contribute to solution quality and parsimony in problem‐solving. The sample comprised 478 six‐ to eight‐year‐olds (219 female) who completed mental rotation, visuospatial memory and gear‐based problem‐solving tasks. In both problem‐solving tasks, spatial skills were associated with solution quality (*β* = .27** or .39**, respectively) and partially with the number of operations (*β* = −.06 or −.16*), indicating higher parsimony. Age was significantly linked to spatial skills and partially to parsimony but not to solution quality. These findings highlight the importance of spatial skills for different aspects of children's STEM‐related problem‐solving.

**Statement of Contribution**

**What is already known on this subject**

- Spatial skills are essential for children's interaction with the environment.
- Spatial skills are related to children's STEM learning.

**What the present study adds**

- The typology of intrinsic/extrinsic and static/dynamic skills represents a coherent framework for investigating spatial processing in STEM problem‐solving.
- Spatial skills are positively associated with solution quality in children's STEM problem‐solving.
- Analyses of tracking data revealed that spatial skills may foster children's problem‐solving efficiency, depending on the specific task requirements.
- The relation between spatial skills and different aspects of problem‐solving abilities may vary across primary school age.

## INTRODUCTION

Understanding the cognitive underpinnings of effective problem‐solving in childhood is crucial for fostering academic and practical skills, particularly in science, technology, engineering and mathematics (*STEM*). Among various cognitive abilities involved in early STEM education, spatial skills play a critical role, yet their influence on problem‐solving remains underexplored (Uttal & Cohen, 2012; Wai et al., 2009). These skills enable individuals to mentally manipulate, organize and reason about spatial relations. Consequently, spatial skills serve as a foundation for interpreting and interacting with the physical world (Hodgkiss et al., 2018), making them one of the most important domains of human intellect (Wai et al., 2009).

The developmental trajectories of spatial skills during childhood are influenced by various factors, including physical activity, environmental interactions and play materials (Borriello & Liben, 2018; Jansen & Pietsch, 2022; Verdine et al., 2014). Research indicates that early activities, such as construction play and digital games, enhance spatial cognition, laying the groundwork for complex skills like mental rotation and visuospatial memory (Polinsky et al., 2023; Shepard & Metzler, 1971).

Despite the established role of spatial skills in supporting mathematical and science learning (Hegarty, 2014; Lowrie et al., 2021), their direct contribution to problem‐solving quality and efficiency in children remains insufficiently understood (Schäfer, Reuter, Karbach, & Leuchter, 2024). This gap is particularly salient in tasks requiring the mental manipulation of spatial information, such as predicting gear dynamics or optimizing configurations in mechanical contexts (Hegarty, 1991; Hegarty et al., 1988; Reuter & Leuchter, 2022). Such tasks may not only rely on spatial skills but also challenge children to balance solution quality with operational parsimony—a skill critical for efficient problem‐solving (Kendall, 2015). To study the application of spatial skills in problem‐solving tasks, the intrinsic and extrinsic dimensions of spatial skills, as categorized by Uttal et al. (2013), offer a nuanced framework.

This study aims to bridge these gaps by examining the interplay between spatial skills, age and problem‐solving performance in 6–8‐year‐olds. By focusing on solution quality and parsimony in gear‐based problem‐solving tasks, we aim to investigate how spatial skills shape children's approach to STEM challenges and how they cognitively develop (Hodgkiss et al., 2021; Uttal et al., 2013).

### Spatial skills

Spatial skills refer to the cognitive abilities that enable individuals to perceive, interpret and mentally manipulate spatial features of objects and relations between them (Mallot, 2024; McNea et al., 2023). Spatial skills can broadly be categorized using Uttal et al.'s (2013) typology: intrinsic versus extrinsic, and static versus dynamic skills. Intrinsic skills involve processing spatial features within a single object, while extrinsic skills concern relations between multiple objects. Static skills refer to resting objects, and dynamic skills involve the processing of movable spatial configurations.

**FIGURE 1:**
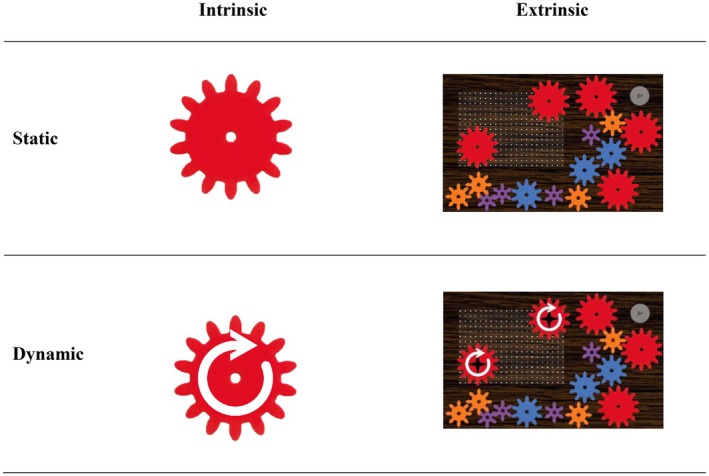
The typology of Uttal et al. (2013), adapted to the spatial features of gears. The images in the four‐field diagram symbolize the intrinsic/extrinsic and static/dynamic properties in the spatial processing of gears.

In the context of gears, Uttal et al.'s (2013) typology provides a coherent framework for understanding how the features and movements of gears and gear configurations are processed. Intrinsic, static skills are used to identify the features of an individual, immovable gear, such as its size and shape (see Figure 1, top left). Intrinsic, dynamic skills involve processing changes in the orientation or location of a single movable gear (Figure 1, bottom left). Extrinsic, static skills come into play when processing immovable gear configurations, allowing for the assessment of distances and relative sizes between gears (Figure 1, top right). Finally, extrinsic, dynamic skills are required to understand the shifting relative locations and turning dynamics of interconnected gears, including their turning direction and speed (Figure 1, bottom right).

Spatial skills comprise memory systems, such as visuospatial working memory and visuospatial short‐term memory (Alloway et al., 2006). While visuospatial short‐term memory temporarily stores spatial information, essential for maintaining accurate spatial representations over short time periods (Nikolova & Macken, 2016; Toornstra et al., 2019), visuospatial working memory actively manipulates spatial information (Baddeley & Hitch, 1974; Gathercole et al., 2004). Another skill enabling the manipulation of spatial information is mental rotation, which describes the ability to identify objects in different orientations (Shepard & Metzler, 1971). Mental rotation can be accomplished by imagining an object's rotary movement but also by alternative strategies, depending on task demands and the individual's cognitive and sensory characteristics (Khooshabeh et al., 2012; Likova, 2012).

Spatial skills are closely associated with attention control in young children (Miller & Simmering, 2016). Uttal and Cohen (2012) even consider spatial skills as a *gatekeeper* for children's STEM learning that is particularly essential for solving challenging tasks. Given the high relevance of spatial skills for learning (Ishikawa & Newcombe, 2021), recent studies have identified training interventions that foster the development of spatial skills, finding that hands‐on exploration was particularly effective (Weber et al., 2024; Yang et al., 2020).

### Development of spatial skills in childhood

Spatial skills begin to develop early in life and may even be innate (Alkouri, 2022). Building on foundational skills such as spatial perception, more complex spatial skills, such as understanding geometric relations and manipulation of visuospatial information, have been shown to develop considerably during preschool and primary school age (Gathercole et al., 2004; Hodgkiss et al., 2018; Möhring et al., 2021). Visuospatial working memory and short‐term memory are critical for learning and education because they enable the retention and processing of spatial information (Cowan, 2014; McAfoose & Baune, 2009). As these memory systems mature, they enable children to perform increasingly complex spatial tasks (Alloway et al., 2006). By middle childhood, many children exhibit notable improvements in tasks requiring both static and dynamic spatial skills, laying the groundwork for success in STEM‐related problem‐solving (Alkouri, 2022; Hodgkiss et al., 2021; Newcombe et al., 2013). A study by Hodgkiss et al. (2021) showed that intrinsic skills, such as understanding the features of individual objects, develop earlier than extrinsic skills, which involve the processing of relations between objects. For example, younger children may excel at identifying and mentally manipulating single objects but struggle with tasks requiring them to coordinate multiple objects systematically.

Children's spatial skills are shaped by everyday experiences, such as interacting with construction toys, playing digital games and exploring physical environments, which can enhance children's understanding of spatial relations (Borriello & Liben, 2018; Jansen & Pietsch, 2022; Polinsky et al., 2023). Construction play is particularly effective in fostering the development of spatial skills (Borriello & Liben, 2018; Verdine et al., 2014; Weber et al., 2024). Moreover, Jansen and Pietsch (2022) found that physical activities were significantly associated with specific spatial skills.

### Problem‐solving: Solution quality and parsimony

Problem‐solving refers to the intentional process of transitioning from an initial state to a desired goal state by applying cognitive and motor resources (Polya, 1957). It encompasses phases of understanding the problem, planning strategies, executing actions and evaluating outcomes (Van Merrienboer, 2013; Viterbori et al., 2017). Solution quality and parsimony are key dimensions of problem‐solving (Kendall, 2015; Phillips et al., 2001; Ward & Allport, 1997).

Solution quality refers to the degree to which a solution meets the predefined goals or criteria of a problem (Jonassen, 2013). For problems with well‐defined goal states, such as gear construction tasks, solution quality is determined by the functional accuracy of the final construction (Schäfer, Reuter, Leuchter, & Karbach, 2024). For example, a high‐quality solution in a gear construction task ensures that all gears mesh correctly and perform their intended mechanical functions (Reuter & Leuchter, 2022). Similarly, in tasks like building a toy bridge, solution quality depends on the structural stability and suitability of the design to span a predefined distance (Shechter et al., 2021). Consequently, achieving high solution quality typically requires an understanding of task‐specific requirements and the ability to translate these into functional outputs (Munoz‐Rubke et al., 2021).

Parsimony, in the context of problem‐solving, refers to proceeding with a minimal number of operations while achieving high solution quality (Ward & Allport, 1997). This efficiency is characterized by a goal‐directed approach that avoids unnecessary actions and redundant iterations. For instance, when designing a gearbox, a parsimonious solution might involve testing only a limited number of gear movements to achieve proper meshing rather than exploring all possible configurations through trial‐and‐error. The process of parsimony relies on using strategies such as systematic testing and optimizing of intermediate solutions. Systematic testing involves identifying operations that are most likely to improve the current state or provide insights into promising next steps (Kendall, 2015; Strimel et al., 2018). For example, when building a toy bridge, a parsimonious approach would focus on using just enough materials to span the river—balancing resource efficiency with the structural requirements (i.e., stability of the bridge; Shechter et al., 2021). In contrast, less advanced heuristics such as trial‐and‐error often lack a clear strategy and rely on arbitrary operations (Polya, 1957). While trial‐and‐error can occasionally lead to successful solutions, it typically results in inefficiency and uncertain outcomes (Funke et al., 2018). For example, randomly connecting gears in a gear construction task may neither improve solution quality nor provide meaningful insights into why certain configurations fail, making the process less parsimonious and less likely to yield high‐quality solutions.

Empirical studies on the interplay of solution quality and parsimony in children aged 5–7 have yielded mixed findings regarding the use of systematic testing and optimizing strategies. Kendall (2015) observed that children often lack systematic failure detection and optimization strategies, as seen in their evaluation of pre‐constructed LEGO bridges. Reuter and Leuchter (2022) found that, while some children in a gear construction task did not engage in testing and optimizing, those who did achieved better solutions. This suggests that, while testing and optimizing might be effective problem‐solving strategies, they are applied inconsistently at this age. Similarly, Lottero‐Perdue and Tomayko (2020) highlighted that children who engaged in systematic testing were more likely to achieve solutions with fewer resources and less effort.

### The role of spatial skills in Children's problem‐solving

Children's ability to systematically test and optimize during problem‐solving tasks may be closely linked to their spatial skills. STEM‐related tasks, like gear construction tasks, often involve understanding complex spatial relations, for example, adjacent gears turn in opposite directions and turning speed depends on gear size (Lehrer & Schauble, 1998; Reuter & Leuchter, 2021). Mental rotation, visuospatial working memory and visuospatial short‐term memory might be crucial for efficient problem‐solving, as they allow children to anticipate dynamic spatial transformations, retain task‐relevant spatial information and adaptively manipulate configurations to achieve goal states parsimoniously.

In the context of gear mechanics, problem‐solving might require understanding the turning direction and speed of interconnected gears. These tasks engage both intrinsic and extrinsic spatial skills, as children must analyse the features of individual gears and the relations between them (cf. Hodgkiss et al., 2018). Spatial skills also enable children to anticipate the outcomes of their actions (Dündar‐Coecke et al., 2020; Möhring et al., 2024), reducing the need for redundant operations. For instance, mental rotation might help children identify potential failures in a gear configuration without testing, supporting both high solution quality and parsimony.

The variability in children's ability to systematically test and optimize may be attributed to developmental factors. During childhood, problem‐solving strategies evolve from unsystematic trial‐and‐error approaches to more goal‐directed and systematic methods (Injoque‐Ricle et al., 2014). Older children are more capable to plan, test and revise their solutions, demonstrating an improved capacity to balance solution quality with parsimony (Lottero‐Perdue & Tomayko, 2020; Strimel et al., 2018). This developmental trajectory reflects the maturation of cognitive control functions, such as cognitive flexibility and working memory, which play a critical role in problem‐solving (Schäfer, Reuter, Leuchter, & Karbach, 2024). Greiff et al. () further emphasize the importance of reasoning abilities, noting that these domain‐general cognitive skills are central to children's performance in complex problem‐solving. However, more research is needed to better understand the factors that contribute to variability in children's solution quality and systematic testing and optimization strategies in problem‐solving, particularly focusing on spatial skills.

### Rationale of the current study

While previous research has established a strong link between spatial skills and STEM learning, few studies have explored how spatial skills specifically contribute to problem‐solving solution quality and parsimony in children. This study aims to address this gap by investigating the relations between spatial skills, solution quality and parsimony in gear‐based problem‐solving tasks. By examining 6–8‐year‐olds, we focus on a critical developmental period for spatial skills and problem‐solving (Hodgkiss et al., 2021; Injoque‐Ricle et al., 2014).

This study includes mental rotation, visuospatial working memory and visuospatial short‐term memory. Mental rotation allows children to imagine spatial dynamics, helping them anticipate object transformations; thus, it is reasonable to assume that this capability also applies to problem‐solving tasks. Moreover, visuospatial working memory enables the active manipulation of spatial information, essential for adapting configurations and retaining intermediate states. Visuospatial short‐term memory describes the capacity to temporarily store spatial information, which is critical for maintaining accurate representations of task‐relevant features. Consequently, the presented study investigates whether these components of spatial skills support systematic problem‐solving, indicated by achieving parsimonious and high‐quality solutions.

### Hypotheses

*Spatial skills are positively associated with solution quality and parsimony*.

*Parsimony is positively associated with solution quality*.

*Age positively affects spatial skills, solution quality and parsimony*.

## MATERIALS AND METHODS

### Participants

A power‐analysis for correlational analyses (parameters: *r* = .20, *α* = .01, 1−*β* = .90) resulted in a required sample size of *n* = 365. A power‐analysis for structural equation model analyses (parameters: *r* = .20, *α* = .01, 1−*β* = .90, *df* = 7) resulted in a required sample size of *n* = 88. A total of 478 students (*M*_age_ = 7.40 years, SD_age_ = 0.64 years; 219 female) from first and second grades in primary schools participated voluntarily and with written informed consent of their parents. They also attended a previously published study, which analysed the relations between executive functions and problem‐solving (Schäfer, Reuter, Leuchter, & Karbach, 2024). Data of two participants were excluded because they aborted participation during the first two tasks. Consequently, 476 datasets remained, meeting the requirements of both power analyses. Inclusion criterion was age (6–8 years). No exclusion criteria were established to obtain a heterogeneous sample. The recruitment period started on 29 November 2022 and ended on 01 March 2023. The study was approved by a local ethics committee.

### Procedure

**FIGURE 2:**
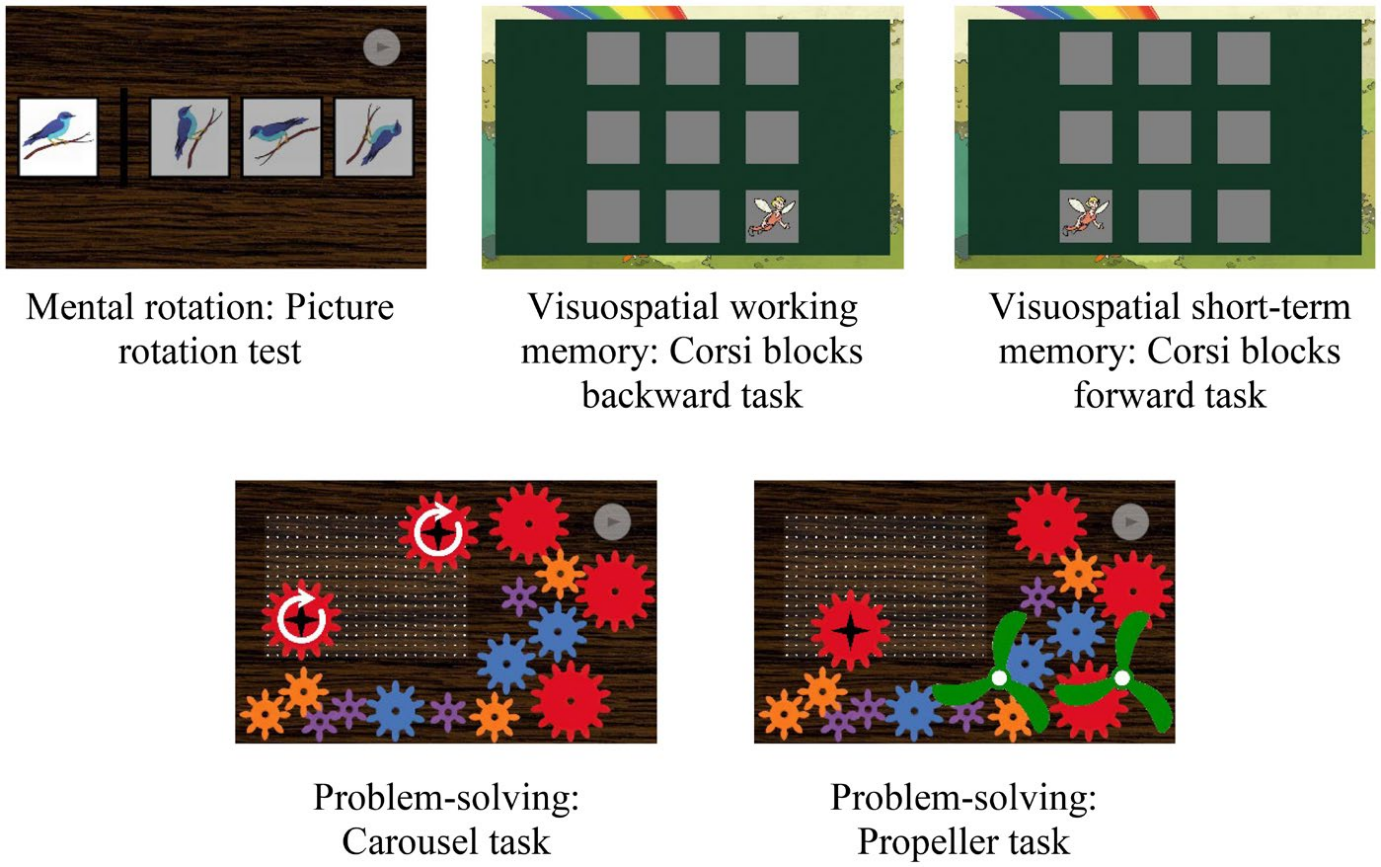
Screenshots of the tasks measuring spatial skills (upper panel) and problem‐solving (lower panel). The tasks were displayed to the participants on tablets.

Participants performed three tasks measuring the spatial skills mental rotation, visuospatial working memory and visuospatial short‐term memory, as well as two gear problem‐solving tasks (see Figure 2). Participants performed all tasks on tablets with instructions provided via headphones. The procedure took approximately 90 min. All tasks were performed under the supervision of trained experimenters using a validated test software (Schäfer, Reuter, Leuchter, & Karbach, 2024). The software tracked all operations, that is, gear *displacements* and *turnings*, in real time as log‐file data. This means that each time a participant shifted a gear to another place on the screen, it was counted as a gear displacement. Each time a participant swiped a gear circularly on the gear‐board, it was counted as a gear turning. How far a gear was shifted or turned was irrelevant for the computation of the operations, meaning that even small movements were registered. The test software conducts preprocessing and data curation automatically, so that solution quality and total numbers of displacements and turnings can immediately be retrieved.

### Tasks

#### Spatial skills—Mental rotation: Picture rotation test (Quaiser‐Pohl, 2003)

One reference image and three selection images were presented two‐dimensionally in a row. All selection images showed the reference image rotated by 45–315°. However, two of the selection images were additionally mirrored. Participants had to pick the selection image that showed the unmirrored equivalent of the reference image. This task consisted of two practice trials followed by 16 test trials (0–16 points).

#### Spatial skills—Visuospatial working memory: Corsi blocks backward‐recall task (Schäfer, Reuter, Leuchter, & Karbach, 2024)

A 3 × 3‐matrix of rectangular squares was presented. In each trial, a fairy appeared on some of the squares sequentially. Participants had to recall this sequence in the reverse order in which the fairy appeared on the squares. The task started with sequences consisting of one visited square that could successively extend up to eight squares. Each square was visited at most once per sequence. For each sequence length, six sequences (i.e., trials) were presented. When more than two of six trials of one sequence length were recalled incorrectly, the task was aborted. This task consisted of four practice trials followed by 6–48 test trials (0–48 points).

#### Spatial skills—Visuospatial short‐term memory: Corsi blocks forward‐recall task (cf. Gathercole et al., 2004)

A 3 × 3‐matrix of rectangular squares was presented. In each trial, a fairy appeared on some of the squares sequentially. Participants had to recall this sequence in the order in which the fairy visited the squares. The task started with sequences consisting of one visited square that could successively extend up to eight squares. Each square was visited at most once per sequence. For each sequence length, six sequences (i.e., trials) were presented. When more than two of six trials of one sequence length were recalled incorrectly, the task was aborted. This task consisted of four practice trials followed by 6–48 test trials (0–48 points).

#### Problem‐solving: Carousel task (Reuter & Leuchter, 2022)

A board was presented, on which two gears were located that could not be moved but turned (i.e., fixed gears). Around the board, 14 different‐sized gears were available to the participants, which they could freely move, connect and turn on the board. The task goal was to connect the fixed gears on the board so that they would both turn in the same direction when one of them was turned. Solution quality was ordinally scaled according to Table 1 (0–4 points). Since the requirements are that particular gears turn in specified directions when they are interconnected, this task mainly focuses on extrinsic spatial features (i.e., the relative turning direction of different gears; Uttal et al., 2013).

**TABLE 1:** Grading system for solution quality in the carousel problem‐solving task.

Coded score	Final state on the gear board
0	Neither the driving gear nor the target gear had another gear connected with it
1	Either driving gear or target gear had at least one other gear connected with it
2	Both driving gear and target gear had at least one other gear connected with it, but driving gear and target gear were not connected with each other
3	Driving gear and target gear were connected with each other, but when the driving gear was turned in the specified direction, the target gear did not turn in the specified direction
4	Driving gear and target gear were connected and when the driving gear turned in the specified direction, the target gear also turned in the specified direction

*Note*: The solution quality of the carousel task is graded according to the final gear configuration a participant has constructed.

#### Problem‐solving: Propeller task (Schäfer, Reuter, Leuchter, & Karbach, 2024)

A board was presented, on which one gear was located, that could not be moved but turned (i.e., fixed gear). Around the board, 14 different‐sized gears were available to the participants, which they could freely move, connect and turn on the board. Additionally, two propellers were located around the board, which could be moved and attached to gears, so that they could turn together. The task goal was to attach the propellers to gears, so that, when the fixed gear was turned, one propeller would turn as fast as possible and the other one as slow as possible, without touching each other. Solution quality was ordinally scaled according to Table 2 (0–5 points).

**TABLE 2:** Grading system for solution quality in the propeller problem‐solving task.

Coded score	Coding variable	Final state on the gear board
0	Turning Speed	At least one propeller was not attached on a gear
1	Turning Speed	Both propellers were attached on same‐sized gears
2	Turning Speed	Both propellers were attached on different‐sized gears, but not the largest and smallest available gears
3	Turning Speed	One propeller was attached on the smallest available gear and the other propeller was attached on the largest available gear
0	Contact	At least one propeller was not driven by the driving gear
1	Contact	Both propellers touched each other when the driving gear turned
2	Contact	Both propellers did not touch each other when the driving gear turned

*Note*: The solution quality of the propeller task is graded according to the final gear configuration a participant has constructed. The solution quality of the propeller task is the sum of the turning speed score and the contact score.

According to Uttal et al.'s (2013) typology, both the carousel and the propeller task require extrinsic spatial processing (carousel: relative turning direction; propeller: relative turning speed). However, the propeller task increases attention on intrinsic spatial object features, such as size and exact location (i.e., gears have to be selected according to their size and propellers should not touch each other). Given its multiple requirements, the propeller task can be considered more complex than the carousel task, and solving it might, thus, require more operations. Consequently, it might be expected that the measure of operations is more discriminative between participants in the propeller task.

In both problem‐solving tasks, participants had a sufficient number of different‐sized gears to construct, test and optimize constructions by moving and turning the task‐relevant objects (i.e., gears and propellers). Within the processing time of up to 3 min, participants were free to carry out as many operations (i.e., displacements and turnings of the gears) as they intended to. However, they were instructed to terminate a task as soon as they were satisfied with their solution to indicate that parsimony was also relevant.

### Statistical analyses

We first computed correlations (Spearman's Rho) between all variables measuring spatial skills, as well as solution quality and parsimony in problem‐solving. Next, we calculated a structural equation model for each problem‐solving task (carousel and propeller). Each of the models included latent factors for spatial skills (indicated by accuracy on the three spatial skills tasks), operations (indicated by the number of gear displacements and turnings, performed in the problem‐solving tasks) and solution quality (indicated according to the performance scores in the carousel and propeller task; see Tables 1 and 2). Notably, fewer operations indicated higher parsimony and vice versa, implying that the latent factor of operations is an inversely coded indicator of parsimony.

## RESULTS

Correlation analyses showed that spatial skills were positively correlated with solution quality and negatively correlated with some measures of operations (i.e., gear displacements and turnings; see Table 3). The correlations were particularly substantial among the measures of operations and among the measures of spatial skills, allowing to merge these measures to common latent factors in the structural equation models, respectively.

**TABLE 3:** Descriptive values and correlation coefficients (Spearman's Rho) between all variables.

Variables	*M*	SD	1	2	3	4	5	6	7	8
1 Mental rotation	8.39	4.48	1							
2 Visuospatial working memory	12.96	6.21	0.36**	1						
3 Visuospatial short‐term memory	17.09	5.62	0.24**	0.44**	1					
4 Carousel task displacements	39.17	23.50	−0.06	−0.07	−0.15*	1				
5 Carousel task turnings	16.94	13.68	−0.10*	−0.12*	−0.08	0.70**	1			
6 Carousel task solution quality	2.85	1.08	0.10*	0.19**	0.16**	−0.03	0.01	1		
7 Propeller task displacements	25.01	15.93	−0.02	−0.02	−0.12*	0.35**	0.24**	0.12*	1	
8 Propeller task turnings	18.16	13.55	−0.01	−0.05	−0.06	0.26**	0.41**	0.10*	0.62**	1
9 Propeller task solution quality	2.24	1.54	0.14**	0.27**	0.29**	−0.04	0.06	0.25**	0.02	0.06

*Note*: The manifest variables Turnings and Displacements represent the number of these operations that were carried out throughout the task.

**FIGURE 3:**
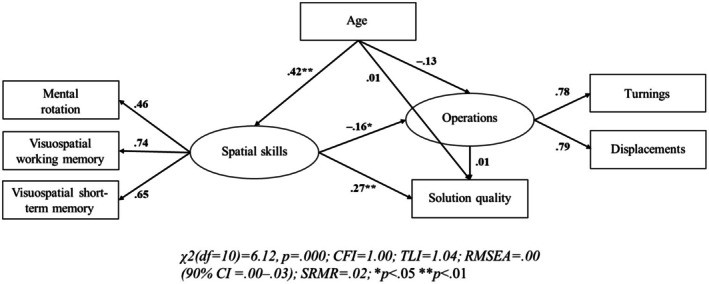
Structural equation model of spatial skills, solution quality and operations in the carousel problem‐solving task. Squares represent manifest variables and circles represent latent factors. Arrows pointing from latent factors to manifest variables represent factor loadings. All other arrows represent regression paths. The manifest variables Turnings and Displacements represent the numbers of these operations that were carried out throughout the problem‐solving task. Thus, the factor Operations is the inversely coded indicator of parsimony.

**FIGURE 4:**
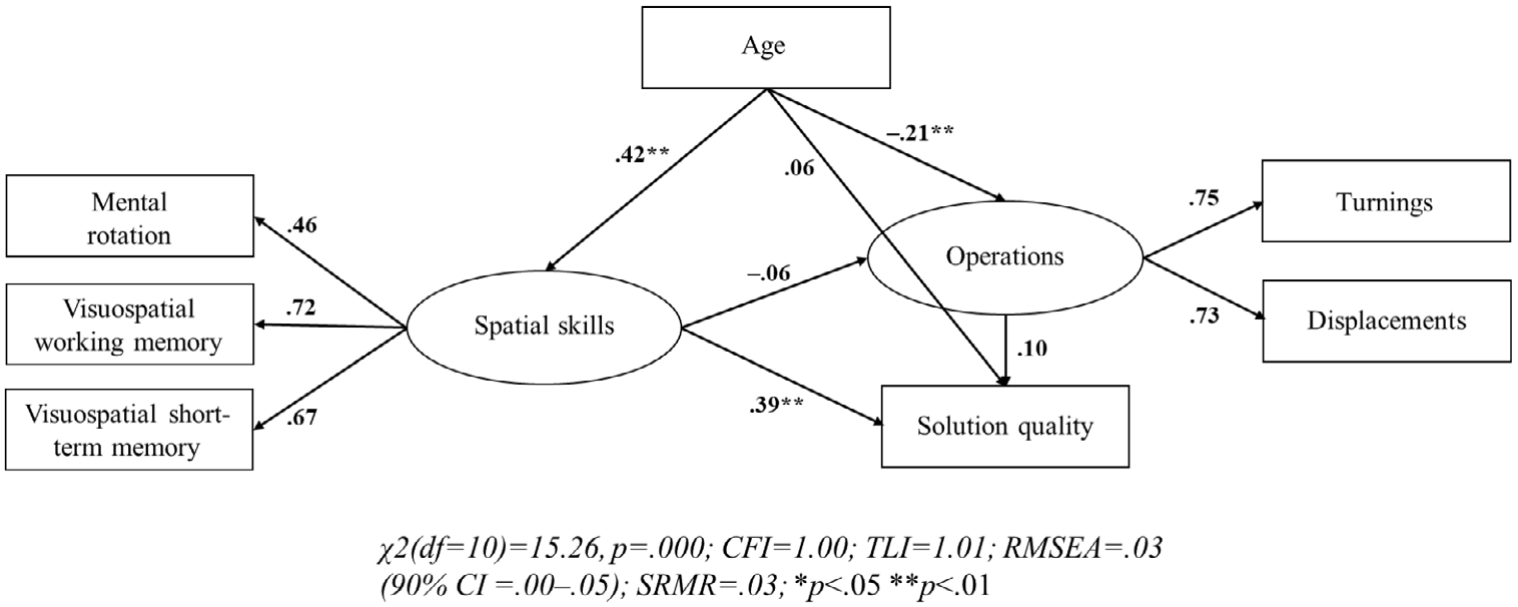
Structural equation model of spatial skills, solution quality and operations in the propeller problem‐solving task. Squares represent manifest variables and circles represent latent factors. Arrows pointing from latent factors to manifest variables represent factor loadings. All other arrows represent regression paths. The manifest variables Turnings and Displacements represent the numbers of these operations that were carried out throughout the problem‐solving task. Thus, the factor Operations is the inversely coded indicator of parsimony.

H1. Structural equation models revealed that spatial skills had a significant positive impact on children's problem‐solving (see Figure 3 for carousel task and Figure 4 for propeller task). Our models revealed that spatial skills had an effect on solution quality in both problem‐solving tasks (*β* = .27** in the carousel task; *β* = .39** in the propeller task). Higher spatial skills were also significantly associated with fewer operations (i.e., higher parsimony) in the carousel (*β* = −.16*) but not in the propeller task (*β* = −.06).

H2. The numbers of operations yielded no significant effects on solution quality in both problem‐solving tasks (*β* = .01 in the carousel task; *β* = .10 in the propeller task; see Figures 3 and 4). This indicates that children conducting few operations were similarly likely to achieve a good solution as children conducting many operations.

H3. Age had a significant association with spatial skills (*β* = .42** in both tasks), but not with solution quality (*β* = .01 in the carousel task; *β* = .06 in the propeller task; see Figures 3 and 4). Furthermore, age had a negative effect on the number of operations, indicating that increasing age led to an increased parsimony, whereas this effect was significant in the propeller (*β* = −.21**) but not in the carousel task (*β* = −.13).

## DISCUSSION

This study examined the relations between spatial skills, problem‐solving quality and parsimony in 6–8‐year‐old children. The findings confirm the crucial role of spatial skills in problem‐solving, in that higher spatial skills were associated with better solution quality and partially with increased parsimony.

H1. The results showed that spatial skills have an important impact on primary school children's problem‐solving. The results align with previous research highlighting the importance of spatial skills in STEM‐related learning and cognitive development (Hodgkiss et al., 2018; Möhring et al., 2021; Uttal et al., 2013). The positive association of spatial skills and solution quality indicates that children who designed a good solution were capable of memorizing locations, sizes and spatial relations between task‐relevant objects (i.e., gears and propellers). This is consistent with prior studies showing that spatial skills enable children to mentally represent and manipulate spatially complex configurations (Shepard & Metzler, 1971; Uttal & Cohen, 2012).

The structural equation models provide closer insights into these relations. The path coefficient from spatial skills to solution quality was stronger in the propeller task than in the carousel task, suggesting that spatial skills played a more prominent role in more complex problem‐solving tasks. This could be due to the additional cognitive demands of the propeller task, which required children to integrate multiple constraints (gear turning speed and propeller contact) rather than a single mechanical relation (gear turning direction). The non‐significant path between spatial skills and operations (thus parsimony) in the propeller task indicates that while spatially skilled children were effective in constructing high‐quality solutions, they did not necessarily complete the task more parsimoniously. This could imply that even high‐ability children engaged in extensive testing and adjustment when tackling challenging tasks.

Since the propeller task strengthened the focus on intrinsic object features (i.e., sizes and locations of gears and propellers), the finding that spatial skills were more dominant in the propeller task solution quality is in line with the finding that intrinsic spatial skills are more developed in 6–8‐year‐olds than extrinsic skills (cf. Hodgkiss et al., 2021). Furthermore, the effects of spatial skills on solution quality are in line with the gatekeeper hypothesis, stating that spatial skills are essential cognitive prerequisites for STEM performance (Uttal et al., 2013; Uttal & Cohen, 2012).

The significant negative relation between spatial skills and operations in the carousel task points to the possibility that children with stronger spatial skills used a more efficient approach, performing fewer unnecessary actions. This aligns with prior findings that indicate spatial reasoning supports mental problem‐solving strategies (Dündar‐Coecke et al., 2020; Möhring et al., 2024). The models highlight an important distinction: While spatial skills contribute to solution quality in both tasks, their impact on parsimony is more task‐dependent, potentially mediated by the complexity of spatial relations involved.

H2. Parsimony, which has been shown to be partially associated with spatial skills, was not significantly associated with solution quality in either problem‐solving task. This finding demonstrates that children using few operations were similarly successful in achieving good solutions as children using many operations, suggesting that they avoided committing redundant operations. The weak and non‐significant paths from operations to solution quality imply that parsimony alone was insufficient to determine problem‐solving success. These findings suggest that developing problem‐solving strategies should not solely rely on minimizing operations but may also integrate broader exploration and structured refinement (Eichmann et al., 2019; Reuter & Leuchter, 2022). This insight aligns with prior research demonstrating that children often engage in iterative hypothesis testing rather than following the concept of efficiency (Akcaoglu et al., 2021).

H3. The structural equation models provide additional insights into the role of age in problem‐solving. In our study, spatial skills considerably improved as a function of age, which is in line with previous findings (Hodgkiss et al., 2021). However, solution quality was not affected by participants' age, which might be surprising in the light of studies showing problem‐solving improvement during middle childhood (Injoque‐Ricle et al., 2014; Schäfer, Reuter, Karbach, & Leuchter, 2024). This suggests that, although older children showed improved spatial skills, this did not translate directly into superior problem‐solving performance. This result might be clarified through closer consideration of the applied problem‐solving strategies across age groups.

Parsimony was significantly higher in older children in the propeller task, but not in the carousel task. This may be due to the greater complexity of the propeller task, making the number of operations a more distinguishing factor. Furthermore, this supports the findings of Hodgkiss et al. (2021), who reported that intrinsic skills, which likely are more important in the propeller task than in the carousel task, mainly develop from 6 to 8 years of age.

### Limitations and future research

While this study provides valuable insights into the role of spatial skills in problem‐solving, several limitations must be acknowledged. The study design did not explicitly assess the children's intentions in conducting certain operations. Future research could complement our study through video‐based qualitative analyses of children's task processing and self‐reports on their intentions (cf. Strimel et al., 2020). Another restrictive aspect is that we assessed problem‐solving in gear tasks only. Confirming the major importance of spatial skills for children's STEM problem‐solving would, thus, require including further materials, for example, building blocks (Shechter et al., 2021), and problem domains, for example, mathematical word problems (Swanson & Beebe‐Frankenberger, 2004).

While spatial skills emerged as significantly associated with problem‐solving, other cognitive factors such as executive functions (e.g., inhibition, cognitive flexibility) were not explicitly modelled. Given their documented relevance to problem‐solving (Schäfer, Reuter, Leuchter, & Karbach, 2024), future studies should explore the interplay between spatial skills and broader cognitive control mechanisms. Future studies should also take into account individual cognitive strategies of spatial processing that have, for instance, been reported in people with visual impairment (Likova, 2012).

Although the study controlled for age, prior experience with mechanical tasks was not assessed. Individual differences in exposure to construction toys or prior engagement with problem‐solving activities could have influenced the results. Future research should investigate how early STEM‐related experiences shape spatial skills and problem‐solving.

The study relied on a cross‐sectional design, limiting causal inferences regarding development. Longitudinal studies tracking changes in spatial skills, cognitive strategies and problem‐solving performance would provide a clearer picture of how these abilities evolve and interact throughout childhood. Future research should thus identify the specific spatial processing mechanisms that children use in problem‐solving, for example, saccadic rehearsal (Pearson et al., 2014), explorative visual search strategies (Bobrowicz et al., 2024) or the spatial integration of interactive objects (Quek & Peelen, 2020). One way to investigate the problem‐solving process is to analyse time sequences of operations in the recorded tracking data to detect behavioural patterns and strategies (Greiff et al., 2015; Hartmann et al., 2022). This may provide insights into how children switch between different problem‐solving phases (Viterbori et al., 2017), which might be particularly interesting to closer specify the prominent role of cognitive flexibility in children's problem‐solving (Schäfer, Reuter, Leuchter, & Karbach, 2024). Moreover, analysing behavioural patterns as temporal sequences may identify whether domain‐specific knowledge is acquired during the problem‐solving process (Alrababah et al., 2024; Greiff et al., 2016). If many participants acquire domain‐specific knowledge during the problem‐solving tasks, this would explain why previous studies barely found effects of domain‐specific knowledge on problem‐solving performance when domain‐specific knowledge was assessed prior to problem‐solving (Reuter & Leuchter, 2022; Schäfer, Reuter, Karbach, & Leuchter, 2024).

Previous studies have indicated that the association of spatial skills and STEM activities might be bidirectional (Hu, 2021; Stieff et al., 2014; Uttal & Cohen, 2012). Therefore, our results give rise to the assumption that gear construction tasks could be a promising instrument to foster spatial skills in pre‐ and primary school children. Hands‐on activities such as construction play, digital games and structured spatial training programmes may be effective in supporting this development (Borriello & Liben, 2018; Lowrie et al., 2021; Verdine et al., 2014). Thus, upcoming studies might conduct gear problem‐solving interventions and analyse their effects on the development of children's spatial skills.

## CONCLUSION

Our study underscores the role of spatial skills for children's problem‐solving in gear construction tasks, particularly with respect to solution quality. Moreover, age and spatial skills were positively related to higher parsimony in problem‐solving. The results suggest that the dimensions of spatial skills, developed by Uttal et al. (2013) that are involved in a problem‐solving task depend on task‐relevant spatial and functional object features. Our study provides new insights into the characteristics of spatial skills and into the cognitive framework of problem‐solving in childhood.

## AUTHOR CONTRIBUTIONS

**Jonas Schäfer:** Conceptualization; data curation; formal analysis; investigation; methodology; project administration; writing – review and editing; validation; software; writing – original draft; visualization. **Timo Reuter:** Conceptualization; investigation; methodology; writing – review and editing. **Miriam Leuchter:** Conceptualization; funding acquisition; resources; supervision; writing – review and editing.

## FUNDING INFORMATION

This work was funded by the Ministry of Science, Further Education, Research and Culture, Rhineland‐Palatinate, Germany (no grant number). The article processing charge was funded by the Baden‐Württemberg Ministry of Science, Research and Culture and the University of Education Schwäbisch Gmünd in the funding programme Open Access Publishing.

## CONFLICT OF INTEREST STATEMENT

The authors declare that there was no conflict of interest.

## Data Availability

The data that supports the analyses presented here are available on request from the first author.
